# Attention‐Deficit/hyperactivity disorder and centralized pain: A review of the case of John F. Kennedy

**DOI:** 10.1002/ccr3.6422

**Published:** 2022-10-08

**Authors:** Satoshi Kasahara, Ko Matsudaira, Naoko Sato, Shin‐Ichi Niwa

**Affiliations:** ^1^ Department of Anesthesiology and Pain Relief Center The University of Tokyo Hospital Bunkyo‐ku Japan; ^2^ Department of Pain Medicine Fukushima Medical University School of Medicine Fukushima Japan; ^3^ Department of Medical Research and Management for Musculoskeletal Pain, 22nd Century Medical and Research Center The University of Tokyo Hospital Bunkyo‐ku Japan; ^4^ Nursing Department The University of Tokyo Hospital Bunkyo‐ku Japan; ^5^ Department of Psychiatry, Aizu Medical Center Fukushima Medical University Fukushima Japan

**Keywords:** attention‐deficit/hyperactivity disorder, celiac disease, centralized pain, irritable bowel syndrome, John Fitzgerald Kennedy, low back pain

## Abstract

John Fitzgerald Kennedy (JFK), the 35th President of the USA, had chronic low back pain deemed to be centralized pain. Reportedly, attention‐deficit/hyperactivity disorder (ADHD) could associate with centralized pain. Based on his biographies, JFK could have had ADHD, a plausible cause of pain that afflicted him.

## Graphical abstract

Patients who tend to be hyperactive, like JFK, have centralized pain, such as chronic low back pain, and may also have comorbid ADHD. Clinicians should screen for ADHD because chronic centralized pain can improve with ADHD medications.

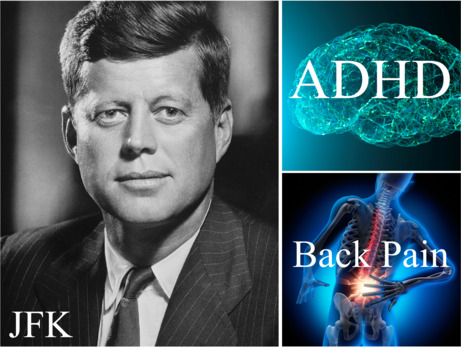

## 1 INTRODUCTION

Lumley et al.^1^ reported on psychotherapy for centralized pain and presented the case of Mr. A, who had chronic low back pain (LBP), a typical case of centralized pain. Mr. A. was a middle‐aged man who had developed LBP 7 years earlier while exercising, without any apparent injury. Although neurological examination revealed no impairment, he had an L4–L5 fusion and later developed a sacroiliac joint fusion; however, he continued to have significant pain. Mr. A.’s parents had high expectations of him. He described himself as a sensitive child who worried that he was not good enough. He was often anxious and had stomach aches before school presentations or due to fear of disappointing his parents, particularly his father. John Fitzgerald Kennedy (JFK, 1917–1963), the 35th President of the United States of America, has a surprisingly similar history to Mr. A. JFK also had chronic LBP, considered centralized pain,^2^ and various other medical issues. He bore unusually high expectations from his parents, especially his father, and was not good at expressing his feelings.^3^

Since childhood, JFK continuously suffered from several diseases, such as irritable bowel syndrome (IBS), malabsorption, adrenal insufficiency, hypothyroidism, chronic prostatitis, allergies, and insomnia.^3^ The most disquieting condition was LBP, which also contributed to his death.^3^ JFK's LBP started in 1937, which developed during football, although a specific cause for his pain was never identified. It aggravated with stress and did not respond to several analgesics, including codeine. Despite undergoing four lumbar spine surgeries, including L4/L5 laminectomy, lumbosacral, and sacroiliac fusion, JFK's LBP exacerbated rather than improved. At the time of JFK's assassination in Dallas in 1963, when the first bullet struck him in the back of the neck, his back brace held him erect, allowing the second and fatal bullet to strike the back of his head.^3^

However, according to the final report of the House Select Committee on Assassinations, other anthropogenic factors contributed to Kennedy's assassination.^4^ Despite being aware of the danger of assassination and warnings from people around him, JFK chose not to attach a protective bubble‐top to the convertible, making it easier for the sniper to take aim. This kind of impulsive tendency was often observed during his early childhood and contributed to his promiscuous behavior even within the White House.^3^, ^5^, ^6^

Attention‐deficit/hyperactivity disorder (ADHD) is a developmental disorder associated with central nervous system (CNS) dysfunction and is classified into predominantly inattentive, predominantly hyperactive–impulsive, and combined types. JFK's biographies are filled with tales of the President's inattention and hyperactivity. In recent years, ADHD has been associated with centralized pain and chronic LBP.^7^, ^8^, ^9^ Moreover, it is believed that JFK had ADHD characteristics.^10^ This article, based on his biographies, provides a literature review of JFK's potential diagnosis of ADHD, and discusses JFK's medical problems, including centralized pain and ADHD.

## 2 CASE HISTORY/EXAMINATION

John Fitzgerald Kennedy was depicted as a lively man, full of energy; he forced himself to act this way. In reality, he suffered from poor health; the details of his ailments remained a secret until 2001 (at the request of the Kennedy family), when the medical archives of the Kennedy library were opened for public scrutiny.

Kennedy's biographer Robert Dallek chose Dr. Jeffrey Kelman, a specialist in internal medicine and physiology, to accompany him, along with Dr. Bert E Park, a neurosurgeon and the author of *The Impact of Illness on World Leaders*,^11^ to examine all records from 1955 through 1963, reading medical reports and several documents. With the help of these records and documents, he reconstructed JFK's clinical history in his biographies.^3^, ^12^ Dallek's biography of JFK has recently been cited in several medical studies.^2^, ^13^, ^14^

James MacGregor Burns was a Democratic nominee in Massachusetts's first congressional district who published a biography of JFK in 1959 to determine whether JFK had the qualifications of a president.^15^ Burns' biography of JFK was written while he was alive and became the presidential candidate, under the condition that Burns would have complete and unrestricted access to his official and personal files, with JFK's consent and assistance from his office and aides. Burns interviewed JFK's wife, parents, family members, teachers, assistants, political supporters, political opponents, and many others. Burns had full access to his files of correspondence, legislative records, family records, and such from the past, as the bibliographical notes explain in greater detail. Burns' biography is based largely on these data and has the most detailed records of JFK's developmental and behavioral characteristics; therefore, it has been cited as an important source of developmental and medical research in the past.^16^, ^17^

## 3 DIAGNOSIS

According to the diagnostic criteria outlined in the Diagnostic and Statistical Manual of Mental Disorders, Fifth Edition (DSM‐5),^18^, ^19^ a diagnosis of adult ADHD is made by at least five of nine symptoms of inattention and/or at least five of nine symptoms of hyperactivity/impulsivity in a person older than 17 years of age. Through our review,^3^, ^15^ we identified episodes in JFK's life that suggest the specific symptoms of inattention and hyperactivity/impulsiveness based on his biographies by Dallek and Burns, which appears to be appropriate for this study.

### 3.1 Observations

Several factors indicate that JFK experienced problems with attention, including inattention related to work‐related activities (DSM‐5 inattention criterion a), difficulty in sustaining attention to tasks (criterion b), being dreamy or preoccupied (criterion c), not following through on instructions in the workplace (criterion d), having difficulty keeping belongings in order and poor time management (criterion e), avoiding tasks requiring sustained mental effort (criterion f), often losing things (criterion g), and frequent forgetfulness (criterion i). Several examples can be quoted as follows: JFK's thesis had “many typographical errors and was English diction defective” (a).^3^ “His lack of diligence in his studies; or, let us say, lack of ‘fight’ in trying to do well in those subjects that didn't happen to interest him….”(b).^3^ “He has the intellectual's type of absent‐mindedness.” He “breaks off a conversation with a staff aide, perhaps in the middle of his own sentence, to reflect for long moments on a different subject” (c).^15^ “He did not feel that he had to live by the ordinary rules governing everyone else. He was always arriving late for meals and classes, setting his own pace, taking the less‐traveled path” (d).^3^ “Jack's sloppiness was symbolic of his disorderliness in almost all of his organization projects.” Jack keeps appointments late. “He was not much for planning ahead” (e).^3^ “Jack studies at the last minute” (f).^3^ “He showed early a trait that baffles his office staff today—an almost photographic memory for correspondence, conversations, and historical fact, but an almost total absent‐mindedness about where he has mislaid speeches, books, and clothing” (g).^15^ “He has even overflowed the bathtub, as was his boyhood custom”.^3^ “He forgets the little things around him because he is preoccupied with what appears to him as bigger ones” (i).^15^

Other descriptions indicate that he also had characteristics of hyperactivity and impulsivity, including often fidgeting with his hands (a), often leaving his place or seeking fast‐paced activity (b), often running about or feeling restless (c), always “on the go” acting as if “driven by a motor” (e), and difficulty in waiting patiently (h). Specifically, as he talked with visitors in his office, Kennedy would fidget with a pencil.^15^ “Kennedy sat tapping his front teeth with his thumb and running his hand through his hair.” “Averell Harriman thought Kennedy was ‘less tense than when I saw him last, but his hands are still constantly in motion’”(a).^3^ He liked madcap drives to get to an airplane or dinner on time (b).^15^ “He hated to waste time; in the morning he would read a magazine while taking a bath and at the same time shave there, guiding his razor by glancing occasionally at a mirror set up on a bathtub tray” (c).^15^ “He was too much in a hurry, that he was going too far too fast, that he should pace himself better, that he should learn to take a breather. But the dynamo would not or could not slow down. He was always in the process of going or coming” (e).^15^ The Congressman hated to be late. “A stop for a train, an unnecessary delay, a buttonholing admirer would tauten Kennedy's face and send him into short tirades back in the car” (h).^15^

Considering the above descriptions of JFK (some of which indicate his mannerisms even before 12 years of age), we believe that JFK met several diagnostic criteria for ADHD outlined in the DSM‐5; these included 8/9 items (a, b, c, d, e, f, g, and i) from the subcategory “Inattention” and 5/9 items (a, b, c, e, and h) from the subcategory “Hyperactivity/impulsivity” in item A of the ADHD section in DSM‐5. This could be considered ADHD of the combined type.

## 4 DISCUSSIONS

To the best of our knowledge, this study is the first report to investigate the possibility of JFK's diagnosis of ADHD in line with valid diagnostic criteria. However, ADHD did not prevent JFK from achieving success because he was able to surround himself with competent, detail‐oriented people (principally, his brother, Robert, who had the exact opposite of JFK's personality), and he was willing to delegate to them both responsibility and authority.^10^

Thus, adult ADHD is less likely to manifest functional impairment when occupational and social demands are met by internal resources, such as intellectual ability and self‐control, and external resources, such as support from family members.^20^ Furthermore, to others, an adult with ADHD is more likely to appear to function normally despite their psychological distress because they invest significant time, energy, and effort or engage in excessive activity to compensate for their functional impairment. Thus, adult ADHD is underdiagnosed in more than 80% of cases, even in psychiatric practice.^20^ Furthermore, since orthopedic surgeons and pain clinicians who treat patients with chronic pain, such as low back pain, are unfamiliar with ADHD diagnosis and management, most ADHD comorbid with pain might be underdiagnosed.^7^

As JFK was also called a dynamo, his “overactivity”, “action‐proneness”, and “ergomania”, which have been described as typical behavioral traits of patients with chronic pain in the previous literature,^21^, ^22^ have been suggested to be undiagnosed ADHD.^9^ Therefore, screening for ADHD in patients with chronic pain is considered important, and ADHD screening instruments such as the Adult ADHD Self‐Report Scale and Wender Utah Rating Scale are widely used in clinical practice and research.^23^, ^24^ However, patients with chronic pain generally tend to deny their psychosocial problems and may underestimate the severity of their ADHD symptoms.^25^ Therefore, the Conners' Adult ADHD Rating Scale,^26^ which consists of two scales, one for patients and one for family members, should be used to screen for ADHD in patients with chronic pain more appropriately.^9^

Pinals et al.^2^ discussed that the presentation of pain, including LBP in JFK, was centralized and could be attributed to CNS dysfunction. CNS dysfunctions that cause centralized pain are assumed to be a common basis for disorders such as myofascial pain, failed back syndrome, fibromyalgia, IBS, and chronic prostatitis, many of which could be identified in Kennedy's medical history.^2^ Among the CNS dysfunctions in centralized pain, the focus of attention in relation to ADHD is the dopaminergic nervous system and prefrontal cortex dysfunction. Dopamine plays a central role in pain perception and descending pain suppression pathways, and reduced dopamine levels may increase pain.^27^, ^28^ Thus, patients with ADHD are assumed to have dopaminergic dysfunction^29^, ^30^ and are considered vulnerable to chronic pain. The prefrontal cortex is also functionally connected to the descending pain inhibitory pathways and can act as a virtual filter to reduce unpleasant stimuli such as pain and itching.^31^, ^32^ Prefrontal cortex performance follows an inverted U‐shaped curve in relation to dopamine and noradrenaline activation and is maximized when concentrations of both transmitters are moderate.^33^ However, because the pathophysiology of ADHD includes impaired dopamine and noradrenaline neurotransmission,^34^ this filter does not function adequately, and ADHD is considered vulnerable to pain. Moreover, the fact that centralized pain can be improved with ADHD medications (methylphenidate and/or atomoxetine) may support the correlation between chronic pain and ADHD.^7^

In addition to pain disorders, conditions such as fibromyalgia, IBS, celiac disease, insomnia, malabsorption, hypothyroidism, and allergies, which were present in JFK, are all physical disorders associated with ADHD.^35^ Given this information, ADHD appears to be a plausible cause of the numerous illnesses that afflicted JFK.

Although the concept of ADHD had not existed during his lifetime, JFK preferred the central stimulant amphetamine for pain management,^3^ which, unintentionally, may have served as self‐medication for his ADHD condition. Moreover, in a previous study,^36^ symptoms in patients with chronic pain, including persistent chronic nonspecific LBP, improved with ADHD medications; it was found that the pain and ADHD symptoms of patients with chronic pain and comorbid ADHD tend to improve with ADHD treatment. The results showed that 35 of 110 patients (31.8%) with chronic pain at various sites, who were referred to a psychiatrist at a pain clinic, were finally diagnosed with ADHD. Of these 35 patients, 21 received adjusted ADHD medications (methylphenidate and/or atomoxetine). Twenty of the 21 medicated patients (95.5%) experienced an improvement in their ADHD symptoms, and 14 of 21 patients (66.7%) experienced a simultaneous improvement in their pain symptoms, as evaluated using the numerical rating scale (NRS). The NRS scores of the 14 patients decreased by 4.6 ± 2.6 points (64.7% ± 30.1%). Moreover, considering that there were only seven patients with persistent chronic nonspecific LBP (among the 21 patients with chronic pain at various sites) who received adjusted medication, 7 of 7 (100%) experienced reduction in pain symptoms, as measured using the NRS (4.3 ± 2.6 points, 65.3% ± 28.2%).

Additionally, Kennedy's son was diagnosed with ADHD,^5^, ^6^ and numerous tragedies have been attributed to thrill‐seeking behaviors in the Kennedy family, suggesting the possibility of genetic ADHD.^5^ On the positive side, such thrill‐seeking behavior increases the likelihood of gaining spectacular success, as demonstrated by the Kennedy family's position in politics.^5^

Furthermore, behind JFK's success, his continued anxiety and fear of rejection from his father, who forced him to become a politician like a surrogate doll, was one of the main conflicts in JFK's life.^3^ As suggested by Lumley et al., JFK's centralized pain would have improved if he had been able to sufficiently resolve conflicts by facilitating emotional processing, such as writing “an unsent letter” to his father.^1^

The limitation of this study is that the ADHD diagnosis of JFK is a hypothetical diagnosis based on the description in the published literature, as the authors did not directly examine JFK.

The President's Panel on Mental Retardation,^37^ organized by Kennedy as one of his frontier policies, contributed to the creation of the term “developmental disability,” which now includes ADHD in the United States Public Law.^38^ After 60 years, the seeds of his ideals have budded and borne findings of a link between ADHD and centralized pain and are about to pave the way for the treatment of centralized pain—a condition that he had suffered from during his lifetime.

## AUTHOR CONTRIBUTIONS

SK and KM conceived the study. SK and NS collected and organized the literature information. SK wrote the manuscript. S‐IN assisted in writing the manuscript. All authors have approved the final manuscript for submission.

## FUNDING INFORMATION

This study was funded by the Japan Society for the Promotion of Science KAKENHI (Grant Number: JP20K07755). The sponsor has no role other than funding.

## CONFLICTS OF INTEREST

The authors declare that there is no conflict of interest.

## ETHICAL APPROVAL

None.

## CONSENT

Informed consent for patient information to be published in this article was not obtained because we have dealt with only already published historical data in this case report. There is nothing in this paper that would harm the human rights of the patient.

## Data Availability

The data supporting the results of this paper are all based on officially published material and descriptions in publicly available biographies.
